# The Bone Marrow Niche in B-Cell Acute Lymphoblastic Leukemia: The Role of Microenvironment from Pre-Leukemia to Overt Leukemia

**DOI:** 10.3390/ijms22094426

**Published:** 2021-04-23

**Authors:** Erica Dander, Chiara Palmi, Giovanna D’Amico, Giovanni Cazzaniga

**Affiliations:** Centro Ricerca Tettamanti, Pediatric Department, University of Milano-Bicocca, Fondazione MBBM, 20900 Monza, Italy; giovanna.damico@asst-monza.it (G.D.); gianni.cazzaniga@asst-monza.it (G.C.)

**Keywords:** B-cell acute lymphoblastic leukemia (B-ALL), microenvironment, bone marrow (BM) niche, pre-leukemia

## Abstract

Genetic lesions predisposing to pediatric B-cell acute lymphoblastic leukemia (B-ALL) arise in utero, generating a clinically silent pre-leukemic phase. We here reviewed the role of the surrounding bone marrow (BM) microenvironment in the persistence and transformation of pre-leukemic clones into fully leukemic cells. In this context, inflammation has been highlighted as a crucial microenvironmental stimulus able to promote genetic instability, leading to the disease manifestation. Moreover, we focused on the cross-talk between the bulk of leukemic cells with the surrounding microenvironment, which creates a “corrupted” BM malignant niche, unfavorable for healthy hematopoietic precursors. In detail, several cell subsets, including stromal, endothelial cells, osteoblasts and immune cells, composing the peculiar leukemic niche, can actively interact with B-ALL blasts. Through deregulated molecular pathways they are able to influence leukemia development, survival, chemoresistance, migratory and invasive properties. The concept that the pre-leukemic and leukemic cell survival and evolution are strictly dependent both on genetic lesions and on the external signals coming from the microenvironment paves the way to a new idea of dual targeting therapeutic strategy.

## 1. B-ALL Is a Genetic Disease

Acute lymphoblastic leukemia (ALL) is the most frequent malignancy in children, representing approximately 25% of all pediatric cancers, with an incidence peak in patients aged 2–5 years. In 85% of cases, childhood ALL is due to the expansion of B-cell precursors (B-ALL) and only 15% to the alteration of T-cell precursors (T-ALL). Pediatric ALL is often characterized by recurrent genetic changes that can affect prognosis. In particular, about 75% cases present chromosomal alterations; some of these confer favorable outcomes (i.e., *ETV6-RUNX1* and high hyperdiploidy), while others are associated with adverse prognosis (i.e., *BCR-ABL1, KMT2A* rearrangements, *TCF3/HLF,* IKZF1 plus and hypodiploidy) [1,2].

In particular, the chromosomal translocation t(12;21) that generates the chimeric fusion gene *ETV6-RUNX1* is the most frequent structural abnormality in childhood B-ALL, which occurs in 20–25% of patients [3].

## 2. The Two-Step Model for Childhood B-ALL: The Pre-Leukemic Phase and the Overt Leukemia

Studies on monozygotic twins with concordant leukemia and retrospective analyses of neonatal blood spots (Guthrie Card) of pediatric patients proved the frequent prenatal origin of some of these genetic aberrations, such as hyperdiploidy, *KMT2A* rearrangements, *BCR-ABL1*, *TCF3-PBX1,* or *ETV6-RUNX1.* The concordance of disease in twins has been explained by the generation of the genetic alteration in utero in one fetus, followed by intraplacental metastasis of the clonal progeny to the other twin. Moreover, the retrospective detection of fusion genes or clonotypic *IGH/TCR* rearrangements already in the Guthrie Card of non-twinned pediatric ALL patients provides direct evidence for a prenatal origin for the majority of B-ALL cases [4,5].

Twin studies have been instrumental in learning other aspects of the natural history of pediatric ALL. Firstly, they showed that the latency period between the prenatal event and the development of overt leukemia is variable, even within a pair of twins, and can be very prolonged, up to about 15 years; indeed, the oldest twin with concordant ALL was 14 years at diagnosis. Secondly, since the concordance rate in twins for disease development is not so high (ranged from 50% or more for children under one year of age characterized by *KMT2A* rearrangements to about 5–10% for older cases), additional genetic events must be necessary to complement the first prenatal event. These additional hits can be various and usually are different between twins and between relapse and diagnosis samples belonging to the same individual, indicating their subsequent and postnatal appearance [6].

**Figure 1:**
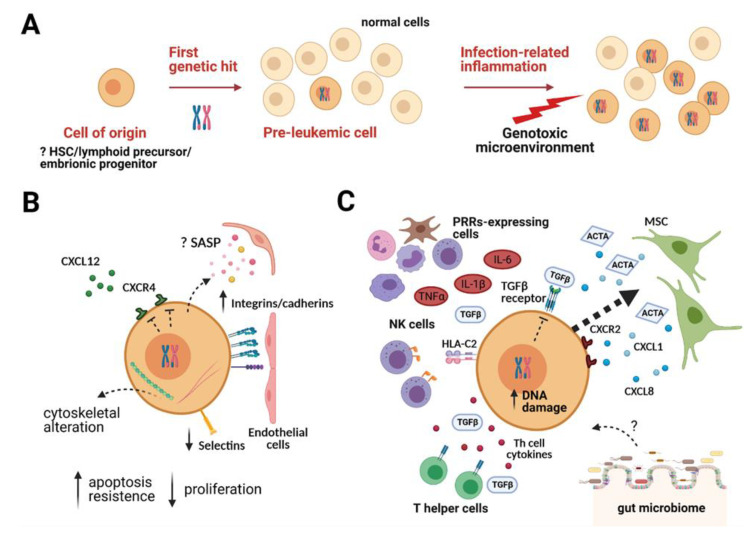
The pre-leukemic cell evolution and its interaction with the microenvironment. (**A**). Model of pre-leukemic evolution to leukemia. The initiating lesion, often a chromosome translocation, occurs in a cell of origin that can differ depending on the first hit and generates a very small clonal population of pre-leukemic cells. Infection-related inflammation is able to create a favorable niche for pre-leukemic cells at the expense of normal cells and promotes their genetic instability, leading to disease manifestation. (**B**) The pre-leukemic cell in the BM niche. Clinical and experimental evidence leads to the hypothesis that pre-leukemic cells are low proliferating and particularly resistant to apoptotic stimuli. The first genetic hit can cause cytoskeletal alterations, dysregulate the expression of adhesion molecules (such as integrins, cadherins, and selectins), and modify cell migration and adhesion properties of pre-leukemic cells (CXCL12-CXCR4 axis impairment and increased ability to adhere to endothelial cells have been reported in in vitro studies). Moreover, pre-tumoral cells can activate a senescence-associated secretory phenotype (SASP, yellow and pink dots) able to influence the surrounding microenvironment (e.g., stimulate endothelial proliferation). (**C**) The pre-leukemic cell in BM niche after an infectious event. We recently showed that the pro-inflammatory cytokines IL-6, TNFα, and IL-1β secreted in response to infections by pattern recognition receptors (PRRs)-expressing cells, cooperate with mesenchymal stromal cells (MSCs) to create a favorable niche for ETV6-RUNX1+ pre-leukemic cells and predisposing them to transformation through increased DNA damage. In this context, MSCs secrete CXCR2 ligands (e.g., CXCL1 and CXCL8, blue dots), attracting ETV6-RUNX1+ cells in a CXCR2-dependent manner (thick dashed arrow), and ActivinA (ACTA), a TGFβ family member. The pre-leukemic cells showed reduced sensitivity to TGFβ and ActivinA–mediated inhibition of proliferation. Furthermore, it has been proposed that T helper (Th) cells can support the growth and survival of pre-leukemic cells, also through the release of cytokines (red dots), including TGFβ. HLA-C2 binding to activating killer immunoglobulin-like receptors (KIRs, orange) on Natural Killer (NK) cells renders them hyporesponsive, thus facilitating immune escape and leukemia development. The gut microbiome is recently considered as a component of the tumor microenvironment, although its role in leukemia transformation is still unclear (“?” in the figure). Created with BioRender.com (accessed on 25 March 2021).

The “minimal” two-step model described above for childhood leukemia, with chromosome alterations frequently providing the first or “initiating” event, implies the existence in the subjects of a pre-leukemic phase that is much more common than the incidence of the overt disease and that only rarely, following the accumulation of secondary events, can clinically manifest in leukemia [3] (Figure 1A).

Several studies have attempted to measure the exact incidence of pre-leukemia in healthy newborns. The results were sometimes contradictory and differed by geographic area and for ALL subtypes [5]. Recently, using the new genomic inverse PCR for exploration of ligated breakpoints (GIPFEL) technique to identify chromosomal translocations at the DNA level, the incidence of the most frequent first hit in pediatric ALL, the *ETV6-RUNX1* fusion, was estimated to be very high: affecting about 5% of healthy newborns, while the incidence of *TCF3-PBX1* fusion was 0.6% [7,8]. In both cases, the frequencies exceeded the ones corresponding to leukemia occurrence (0.01% for *ETV6-RUNX1+* ALL and ∼0.002% for *TCF3-PBX1+* ALL).

Therefore, unraveling the key molecules and the players responsible for the long persistence of the pre-leukemic cells in individuals and for their eventual transformation into cancer cells is fundamental for their eradication and would have many important clinical implications.

## 3. The Pre-Leukemic Cell and Its Interaction with the Microenvironment

### 3.1. The Pre-Leukemic Cell

Since the pre-leukemia phase is usually clinical silent, it is very difficult to study these cells and their characteristics. Exceptions are twins in which one sibling develops the disease, while in the other, apparently healthy, a pre-leukemic phase persists, or a small subgroup of children with ALL who have a previous preclinical phase of transient aplasia that precedes ALL by approximately 2–9 months and heals spontaneously or is very responsive to corticosteroids. Immunoglobulin or T-cell receptor gene analysis confirmed the presence of pre-leukemic cells in the peripheral blood (PB) of the healthy twin and in these very few cases of children in aplastic phase [9].

Because of the above-mentioned difficulties, the identity of the pre-leukemic cell is often unknown. It has been speculated that the cell of origin might be different depending on the first hit. For example, *KMT2A* and *BCR-ABL1* rearrangements probably occur in CD34+ CD19- stem cells [10], while other translocations seem to arise in more differentiated lymphoid-committed precursors [11]. In addition, the expression of the genetic lesion might cause the manifestation of an aberrant phenotype, as in the case of *ETV6-RUNX1* fusion in which some authors hypothesize that the pre-leukemic cell has a peculiar CD34+ CD38-/low CD19+ phenotype that is not detectable in normal bone marrow (BM) [9]. It has also been postulated that the oncogene may arise in a very stem cell and cause the reprogramming of its epigenetic status. These epigenetic changes are then inherited by the daughter cells and maintained during differentiation [12,13]. Instead, Böiers et al. have supposed that *ETV6-RUNX1* may occur in a progenitor unique to embryonic life [14].

Whatever the identity of the pre-leukemic cells, they consist of a very small clonal population (Figure 1A). In the case of the *ETV6-RUNX1* pre-leukemic cell, it is believed that its frequency in positive subjects is about 10^−4^% (several studies reported values from 10^−3^ to ≤10^−5^) [5]. This low frequency makes their identification and characterization even more challenging.

It has been observed that children may relapse after a long period of remission because of the emergence of a leukemic clone evolved from one pre-existing at diagnosis; therefore, in these patients both diagnosis and relapse clone originate from an identical pre-leukemic cell. This observation suggests that the pre-leukemic cell compartment may persist even when the cancer cells have been effectively eradicated by the therapy and that the relapse results from the occurrence of a second independent hit in the same pre-leukemic cell [15]. Therefore, it is conceivable to think that the pre-leukemic cell could be resistant to conventional therapies, probably as an effect of its low-proliferating condition and/or increased resistance to apoptotic stimuli (Figure 1B).

In accordance with this hypothesis, in vitro experiments based on the expression of *ETV6-RUNX1* in cell lines demonstrated its negative impact on cell proliferation [16,17] and its ability to increase the levels of anti-apoptotic molecules such as heat-shock proteins, survivin, and hsa-mir-125b-2. The latter is a cluster consisting of three miRNAs that provide a survival advantage to a broad spectrum of growth-inhibitory signals through inhibition of caspase-3 activations [18,19,20]. Moreover, Hong D. et al. showed that *ETV6-RUNX1* enhances resistance to Fas-L and to chemotherapy drugs such as camptothecin and melphalan [9].

Besides an intrinsic, genetically determined survival advantage to apoptotic signals typical of the pre-leukemic cell, the localization and the interactions with the microenvironment are certainly crucial in regulating its quiescence and maintenance.

Since the pre-leukemic cell, as discussed above, is a stem cell or a B-cell precursor (BCP), it is expected to be resident in the BM, although a direct demonstration of its ability to leave the BM, despite its immature status, is represented by its detection in the peripheral blood (PB) at birth (in Guthrie cards, cord blood, and PB) [6,9,21].

### 3.2. The Bone Marrow (BM) Microenvironment

The BM is an organ with a complex structure, vascularized, innervated, and contained in the bone. It hosts several cell types, both hematopoietic and non-hematopoietic cells. The non-hematopoietic cells belong to different cell lineages, including osteoblasts, osteoclasts, adipocytes, reticular cells, endothelial cells, smooth muscle cells, mesenchymal stromal cells (MSC), and cells of the sympathetic nervous system [22]. These cells not only physically surround hematopoietic cells but actively regulate hematopoietic processes through the secretion of cytokines, hormones, and growth factors and the expression of receptors and adhesion molecules [23]. Although they are a rare population, MSCs are key BM elements. They are fibroblast-like cells with the multipotent ability to differentiate, under appropriate stimuli, into cells of the three mesodermal lineages: osteocytes, adipocytes, and chondrocytes. MSCs include different cell subsets characterized by different localization, expression of specific antigens, and secretion of different molecules [24].

In addition to cellular components, the extracellular matrix (ECM) is another important factor. It comprises more than 200 proteins (fibronectin, collagens, laminin, proteoglycans, growth factors, cytokines, and enzymes) continuously secreted by the different cells for which they, in turn, provide anchorage and regulate their functions [22].

The hematopoietic stem cell (HSC) niche concept was first proposed by Schofield in 1978 as the surrounding environment of HSCs [25]. To date, at least three distinct HSC niches have been widely described: endosteal, arteriolar, and sinusoidal. Each niche has defined characteristics, distinct cellular types, and a peculiar oxygen tension [22].

Osteoblasts localize in the inner surface of the bone, the endosteal surface, and they are considered the key orchestrators of the endosteal niche. They negatively regulate HSC proliferation and stimulate erythroid differentiation by the production of osteopontin and erythropoietin (EPO). These cells were among the first identified as key regulators of HSC maintenance. However, Kiel et al. showed that only 14% of HSCs localized to the endosteum, while most of them localized to arterioles and sinusoids [26]. The C-X-C motif chemokine ligand 12 (CXCL12), expressed by osteoblasts, was demonstrated to be responsible mainly for early lymphoid progenitor retention [27].

Arteriolar niches are populated by several different stromal cells, endothelial cells, sympathetic nervous system nerves, and non-myelinating Schwann cells [22]. CXCL12-abundant reticular (CAR) perivascular stromal cells, an MSC subpopulation, have been recognized as important regulators of HSC maintenance and differentiation. Furthermore, the neural-glial antigen 2 (NG2)-positive periarteriolar cells have been demonstrated to promote their quiescence and maintenance in the BM [28]. Arterial blood vessel endothelial cells, thanks to their low vascular permeability, maintain HSCs in a low reactive oxygen species (ROS) state [29]. Therefore, this niche promotes HSC quiescence and protects them against genotoxic insults [28].

Sinusoidal vessels are more permeable than arteriolar vessels and are instead thought to promote HSC activation. Higher ROS levels in HSCs cause cell-cycle activation and increase migration and differentiation capacities [29]. The sinusoidal niche is therefore the place of the traffic of leukocytes in and out of the BM and a proliferative milieu.

It is believed that there is a continuous exchange between the arteriolar and sinusoidal niche and that the HSC pool is in the balance between proliferation, trafficking, and quiescence [28].

Importantly, the entire BM microenvironment, including all three above-described niches, displays low oxygen pressure. The BM hypoxia signaling is a central element regulating HSC quiescence and metabolism. Surprisingly, recent direct in vivo measurements of local oxygen tension in the BM of living mice have overturned previous hypotheses and shown that the endosteal region is the region with the highest pO2, while the less oxygenated are the deeper peri-sinusoidal regions [30].

In addition to physical and non-hematopoietic elements, several hematopoietic cells in the BM influence the behavior of HSC: for example, megakaryocytes are physically associated with HSCs, regulating their quiescence or proliferation [31], macrophages participate in the regulation of HSC release into the circulation [32] and dendritic cells control HSC trafficking by regulation of sinusoidal vascular permeability [33].

### 3.3. The Pre-Leukemic Cell in the BM Niche

Several studies investigated whether the leukemic stem cells reside in the same BM niches as wild-type cells and how much they are dependent on the signals from stroma cells, but very little is known about the localization and interactions of the pre-leukemic cell with the BM microenvironment.

In this regard, we showed, in two in vitro models of ETV6-RUNX1+ pre-leukemia, that the fusion gene is able to cause alterations in the expression of cytoskeletal regulatory genes and migration properties of BCPs [34].

In detail, we demonstrated that *ETV6-RUNX1* induction in murine interleukin-3 dependent pro-B Ba/F3 cell line dysregulates the expression of several adhesion molecules (CD44, CD11a, CD54, CD18, and CD29) and, as a result, the ability of the pre-leukemic cells to adhere to murine endothelial cell lines is increased. We also showed that this aberrant transcription factor alters the expression of genes regulating cell shape, pseudopodia formation, cytoskeletal dynamics, including actin/microtubule organization and cell migration. Among the most overexpressed genes in ETV6-RUNX1+ cells, we identified *cell division cycle 42* (*CDC42*), a gene coding for a protein involved in cell-cycle progression and in cytoskeleton rearrangement occurring during directional migration. Consistently, the fusion gene significantly impaired the chemotactic response to CXCL12, a key chemokine for HSC and BCP localization. We described that this defect is due to a block in CXCL12-CXC chemokine receptor 4 (CXCR4) signaling axis, while the expression of the receptor is unaffected [34].

Interestingly, alterations in the expression of adhesion molecules and defects in the migration to CXCL12 have also been described following the expression of the fusion gene *BCR-ABL1* in myeloid cell lines. In particular, it has been reported that *BCR-ABL1* diminishes the expression levels of the adhesion molecules P-selectin glycoprotein ligand-1 (PSGL-1) and L-selectin, causes loss of responsiveness to CXCL12, modifies the phosphorylation of several focal adhesion proteins with alteration of integrin function and reduces actin polymerization, causing alterations in cellular morphology [35,36,37,38].

Moreover, gene expression studies that compared *KMT2A*-rearranged with non-*KMT2A*-rearranged infant ALL cases identified in *KMT2A*-rearranged samples the up-regulation of genes involved in homophilic cell adhesion (in particular protocadherin (*PCDH*) *γ* subfamily of genes). It has been assumed that the overexpression of these genes may cause different cell migration and invasion properties of *KMT2A*-rearranged cells [39].

It is, therefore, clear that the first genetic hit in HSC or BPC can change its adhesion/migration properties. This mechanism could be crucial for the long life of the pre-leukemic clone in some BM niches and for its competition for survival signal with normal cells, although there are no studies that have directly verified this hypothesis studying the pre-leukemic cell in situ in its niche. This knowledge will be fundamental for devising strategies for its eradication. For example, the observation that *BCR-ABL1* modifies the expression of some integrins led to testing antibody-mediated selectin blockade as a novel strategy to prevent homing and engraftment of BCR-ABL1+ cells [38].

Another important question still open is whether the pre-leukemic cell, despite being at low frequency in the individual, can modify the microenvironment. In support of this hypothesis, there is evidence that not only leukemic cells, but also normal HSC plays an active role in the niche. In fact, in addition to secreting molecules with autocrine activity, HSC also produces factors capable of modeling the microenvironment: proteins important for angiogenesis and vascular remodeling such as vascular endothelial growth factor (VEGF), angiopoietin 1 (ANGPT1) and bone morphogenic proteins (BMPs) 2 and 6 that induce MSCs to differentiate into mature osteoblasts. Moreover, it stimulates the production by osteoblasts of leukemia inhibitory factor (LIF) and interleukin-6 (IL-6), which, among other functions, promotes osteoclastogenesis [40].

2D and 3D in vitro cell cultures and in vivo mouse models mimicking pre-leukemic BM niche will allow us to understand if the pre-leukemic cell is able to modify the microenvironment in the same way as a normal or leukemic cell does or in a different and peculiar way.

It is also known that the expression of an oncogene in a cell in the pre-tumoral phase can give rise to a senescence mechanism called oncogene-induced senescence (OIS). This premature senescence is not entirely irreversible and does not cause telomere shortening [41]. One of the characteristics acquired by senescent cells is the ability to release a series of factors, including proteases, growth factors, cytokines, with a powerful paracrine and autocrine effect. These molecules constitute the senescence-associated secretory phenotype (SASP), and they are able to influence the surrounding microenvironment [42]. Elements of the SASP are, for example, VEFG, CXC chemokine ligand 8 (CXCL8), CXCL1, and IL-6 that have been shown to stimulate endothelial cell proliferation and contribute to angiogenesis. Furthermore, SASP factors including CXCL1, C-C motif chemokine ligand 2 (CCL2), and ECM-degrading enzymes have been reported to recruit immune cells around senescent cells with the function of eliminating them. However, some senescence cells can express inhibitory ligands creating an immunosuppressive microenvironment and thus promote the escape of fully transformed cells from immune surveillance. Finally, extracellular vesicles (EVs) from senescent cells have been reported to take part in the SASP signature. Indeed, they act as signaling molecules that transfer premature senescence phenotype to neighbor cells and modify recipient cells as SASP factors do [43] (Figure 1B).

## 4. The Role of the Microenvironment in the Transition from Pre-Leukemia to Overt Leukemia

The etiology of the onset of pediatric leukemia has been the subject of many epidemiological and biological studies. Although many environmental exposures have been investigated for a causal role in leukemia development, infection or immune responses to infections are the most accredited triggers to promote pre-leukemia to leukemia transition [4]. In particular, it has been postulated that an abnormal immune response to infections plays a crucial role in this transition [4]. Moreover, inhibition of inflammatory signaling in a selected genetical context (PAX5 mutant cells) has been proved to mitigate B-cell leukemogenesis [44].

Literature data suggest that immune deregulation could be already present at birth. Some reports showed differences in the concentration of inflammatory cytokines in the neonatal blood of children that developed leukemia later on, compared with controls, and that this cytokine signature could be influenced by several characteristics, such as gestational age and sex [45,46].

On the other hand, it has been hypothesized that exacerbated immune response may be the paradoxical consequence of a lack of exposure to infections in early childhood [4]. Support to this hypothesis has come from epidemiological studies demonstrating that children with an under-exposed immune system during infancy have an increased risk of developing leukemia, possibly due to an over-reaction of their uneducated immune system to late-onset childhood infections [4]. In addition, recent studies on pre-leukemic murine models showed that animals born and kept for one month in a specific pathogen-free (SPF) environment, after exposure to a common infectious environment, developed B-ALL, although with low penetrance [13,47].

Notably, a specific pathogen responsible for this process has not been identified, but evidence suggested that infection-related inflammation causes profound changes in BM niches, transforming them in leukemia-favoring microenvironments [48].

Accordingly, we showed that transforming growth factor β (TGFβ), a pleiotropic cytokine produced during inflammation by several stromal and immune cells [49] and activin A, another member of the TGFβ family secreted by MSCs after exposure to pro-inflammatory stimuli, favored the emergence of ETV6-RUNX1 pre-leukemic cells over their normal counterparts in competitive growth assays [16,50]. In brief, we demonstrated that the fusion gene associates with the intracellular signal transducer SMAD3 preventing its signaling in the case of TGFβ/activin pathway activation [16]. As a consequence, while these two cytokines inhibit the proliferation of normal progenitor B cells, they have minor effects on the pre-leukemic ETV6-RUNX1+ cells, thus facilitating the competitive expansion of the latter [16,50].

Mesenchymal contribution to leukemia has been mainly investigated in the context of covert disease, while very less is known about the role of BM-MSCs in the transition from latent pre-leukemic phase to overt disease. Regarding this, two studies conducted on myeloid pre-leukemic conditions showed that perturbations in the stromal microenvironment can lead to hematological malignancies [51,52]. In particular, the deletion of DICER1, an RNase III endonuclease responsible for miRNA biogenesis, in mesenchymal osteoprogenitors causes a hematopoietic disorder that recapitulates the human myelodysplastic syndrome, including the development of acute myeloid leukemia (AML) [51]. Instead, by using a murine model of the Shwachman–Diamond syndrome (SDS) pre-leukemic disorder, specific inflammatory signals from BM-MSC (S100A8/9-TLR4 signaling) were shown to induce genotoxic stress in HSCs leading to leukemic evolution [52].

Recently, we showed that the pro-inflammatory cytokines IL-6, tumor necrosis factor α (TNFα) and IL-1β, secreted in response to several types of infections [53] by pattern recognition receptors (PRRs)-expressing cells, cooperate with BM-MSCs in creating a favorable niche for ETV6-RUNX1-expressing cells and predisposing them to transformation through increased DNA damage [17]. In particular, we showed that inflamed MSC niche strongly decreases proliferation and survival of control cells but not of pre-leukemic Ba/F3 cells, potently attracts ETV6-RUNX1+ cells in a CXCR2-dependent manner, and favors leukemic progression by increasing both the extent of DNA damage and the activation-induced cytidine deaminase (AID) gene expression, a mutagenic enzyme crucial for antibody diversity [17].

All these observations provide a strong rationale for the therapeutic targeting of inflammatory signaling in the pre-leukemic BM niche.

In addition to stromal cells, the immune microenvironment may also play an important role in the transition from pre-leukemia to leukemia. In particular, it has been proposed that memory T helper cells can support pre-leukemic BCP [54] and that Natural Killer (NK) cells with a low killing efficiency can confer a higher risk of ALL development by failing to clear pre-leukemic cells and early leukemic progenitors [5,55].

Finally, recent studies showed that not only pathogens but also commensal microbes, which live in the human body, in particular in the gut, could affect a large set of pathophysiological processes. Changes in the composition and proportion of the gut microbiome can influence the function of distant organs, promoting the onset of several non-oncological and oncological diseases. For these reasons, the microbiome is nowadays considered an important component of the tumor microenvironment [56]. This aspect is particularly important for the evolution from pre-leukemia to B-ALL. It has recently been shown in murine models of B-ALL that disruption of the microbiome by antibiotic administration in pre-leukemic mice in the first weeks of life was sufficient to induce leukemia [57]. Although it is not yet clear what are the mechanisms underlying the interplay between microbiome, pre-leukemic cell, and tumor transformation, these studies open new scenarios on the possibility of acting on the microbiome to develop effective strategies for preventing the development of overt leukemia (Figure 1C).

## 5. The BM Niche in Overt B-ALL: A Corrupted Microenvironment Reprogrammed to Sustain Leukemic Cells

**Figure 2:**
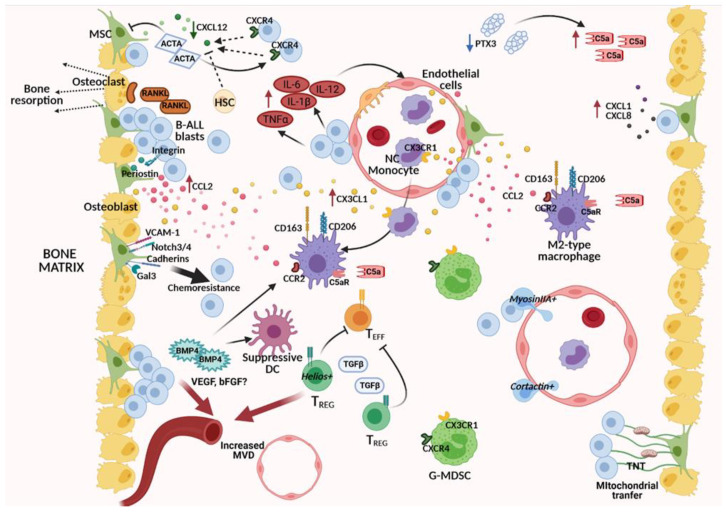
Leukemia-induced alterations within the B-ALL BM niche. B-ALL cells modify the BM microenvironment by cross-talking with MSCs, vascular endothelial cells, osteoblasts, and osteoclasts. Left part, from top to bottom: MSCs produce high levels of Activin A (ACTA). Activin A down modulates (green down arrow) CXCL12 production (green dots) by MSCs, promotes CXCL12-driven migration of B-ALL cells while decreasing healthy HSC migration in response to CXCL12. B-ALL blasts, through RANKL secretion, can activate osteoclasts, causing bone resorption. The interaction of B-ALL cells with MSCs through a periostin/integrin pathway induces the release (red up arrow) of CCL2 (pink dots). Upon contact, MSCs can promote chemoresistance in B-ALL cells through VCAM-1, Notch3/4, cadherins, and Galectin (Gal)3-dependent pathways. BMP4 is another TGFβ family member abundantly produced by leukemia-conditioned MSCs, which contributes to shaping the BM immune microenvironment: it can promote the generation of suppressive dendritic cells (DCs) and the polarization of M2-type macrophages. Increased microvessel density (MVD) is a typical feature of the B-ALL niche, possibly as a consequence of vascular endothelial cell-mediated neoangiogenesis in response to pro-angiogenic stimuli, including VEGF and bFGF. Central part, from top to bottom: The BM microenvironment is rich (red up arrow) in inflammatory mediators such as IL-6, IL-12, IL-1β, and TNFα, in part directly secreted by B-ALL blasts themselves. CX3CL1 (yellow dots), highly represented (red up arrow) in the B-ALL BM, can be secreted by endothelial cells stimulated by inflammatory mediators and by MSCs. Non-conventional (NC) monocytes, abundant in the PB, are possibly recruited through the CX3CR1/CX3CL1 axis into the BM and can differentiate in CD163+/CD206+ M2-type macrophages under the polarizing action of CCL2, C5a, and BMP4. Expansion of immunosuppressive granulocyte-myeloid-derived suppressor cells (G-MDSC) and Helios+ regulatory T cells (Treg) further define the B-ALL BM immune infiltrate and, by secretion of TGFβ can contribute to inhibit effector T cells, while positively impacting on angiogenesis. Right part from top to bottom: Decreased levels (blue down arrow) of PTX3 possibly contribute, by unleashing Complement cascade activation, to high C5a levels (red up arrow). CXCL1 (purple) and CXCL8 (gray dots) are abundant (red up arrow) in the B-ALL BM and can be secreted by MSCs interacting with B-ALL cells. CCL2, produced by leukemia-conditioned MSCs and by endothelial cells stimulated by inflammatory mediators, can recruit macrophages and, in concert with C5a, can induce their M2-like polarization. B-ALL cells expressing high cortactin and myosin-IIA levels can extravasate into blood vessels and disseminate in response to chemotactic stimuli to generate extramedullary metastases. MSCs further support B-ALL cells by transferring mitochondria by means of tunneling microtubes (TNT). Created with BioRender.com (accessed on 23 March 21).

In the last few years, a growing body of evidence is clearly indicating that a “niche effect” is contributing and profoundly impacting leukemic cell proliferation, survival, resistance to therapy, and invasiveness, independently from patient-specific genetic lesions. Leukemic cells interact with the surrounding BM microenvironment in a very active way, reprograming stromal, vascular components, and even immune cells to become leukemia permissive or even supportive by changing their adhesive, secretive, and metabolic properties (Figure 2). Indeed, the identification of recurrent altered molecular pathways in the leukemic BM niche is pivotal to identify new therapeutic strategies targeting the leukemic microenvironment to be coupled with direct anti-leukemic drugs.

### 5.1. Altered Molecular Pathways in the Cross-Talk between BM Stroma and B-ALL Cells

As described above, BM-MSCs represent key stromal components in the maintenance and regulation of healthy hematopoiesis. Recent reports highlight a bidirectional cross-talk between BM-MSCs and leukemic cells crucial for the generation of a leukemia-supportive microenvironment. Cytogenetical analysis of MSCs isolated from the BM of B-ALL patients failed to identify chromosomal alterations typical of the leukemic cells in the majority of cases [58]. *KMT2A-AF4* was the only fusion gene documented in BM-MSCs from KMT2A-AF4-positive B-ALL cases, suggesting that this specific mutation could arise in pre-hematopoietic precursors [59]. Despite the lack of genetic abnormalities, MSCs isolated from the BM of B-ALL patients at disease diagnosis (ALL-MSCs) showed reduced proliferative capacity and ability to sustain long-term hematopoiesis [58,60]. Furthermore, accumulating evidence indicates that B-ALL cells can “corrupt” BM-MSCs to become leukemia-supportive by modulating their cytokine and chemokine release and expression of adhesion molecules, as described below.

#### 5.1.1. Deregulated Chemokine Axes

In a physiological BM niche, stroma-derived chemokines are important signals for regulating hematopoiesis. As already mentioned, CXCL12, secreted by BM-MSCs, plays an essential role in maintaining the quiescent BM hematopoietic stem cell pool. Independent studies on BM plasma samples of pediatric B-ALL patients at disease diagnosis have demonstrated a dramatic decrease in this chemokine [61,62,63]. Alongside, ALL-MSCs have been shown to secrete significantly lower CXCL12 compared to MSCs isolated from healthy donors (HD-MSCs) [64]. Several reports indicate that leukemic cells can specifically migrate in response to MSC-derived CXCL12 in an amount-specific manner and that this migration can be inhibited by the CXCR4 antagonist AMD3100 [62,65,66]. Even if B-ALL cells in vitro co-cultured with HD-MSCs did not directly modulate CXCL12 secretion by MSCs [62], our group recently demonstrated that Activin A, a soluble mediator belonging to the TGFβ family, overexpressed in the B-ALL BM niche [63], is able to downregulate CXCL12 production by BM-MSCs [50]. Moreover, Activin A was demonstrated to significantly enhance in vitro the migration of B-ALL cells in response to CXCL12, even if present at very low doses, while inhibiting the migration of healthy CD34^+^ cells [63]. Indeed, this molecular pathway could be relevant for leukemic cells to gain strict contact with the supportive BM stromal niche, at the expenses of healthy hematopoietic cells.

Beyond the CXCR4-CXCL12 axis, other chemokine pathways resulted significantly altered in the leukemic BM niche. Ex vivo experiments from different research groups indicate that leukemia stimulation of primary BM-MSCs is able to specifically upregulate the expression of CCL2, CXCL10, CCL22, CXCL8, and CXCL1 [67,68,69,70]. Chemokine level analyses in the BM plasma of independent cohorts of B-ALL patients and healthy donors (HDs) further validated some of these results, as CCL2, CXCL1 and CXCL8 were significantly increased in leukemic patients at disease diagnosis and relapse but were comparable to HDs at disease remission [62,67,70]. Interestingly, these chemokines, abundant in leukemia-conditioned MSC supernatants, were able to potently attract B-ALL cells that, besides CXCR4 [63,71], showed high membrane levels of CCR4 (CCL22 and CCL2 receptor) [62] and variable levels of CXCR1/2 (CXCL1 and CXCL8 receptors) [72]. The chemotaxis of healthy hematopoietic progenitors resulted oppositely regulated, as their migration toward MSC-leukemia co-cultures resulted severely decreased compared to MSCs alone [62]. Concerning CCL2 overexpression within the leukemic niche, Ma and colleagues [69] recently demonstrated that the production of this chemokine can be finely tuned by the interaction of leukemic cells with BM-MSCs in a cross-talk mediated by periostin, a matricellular protein with several functions, including osteology and tissue repair. In detail, MSC-derived periostin stimulates, through integrin binding, CCL2 production and release by B-ALL cells, that in turn stimulates periostin expression in BM-MSCs in a sort of self-reinforcing loop [69]. As demonstrated in a periostin knock-out leukemia mouse model, this molecular pathway resulted crucially involved in leukemia adhesion to MSCs, proliferation and dissemination [69,73].

In addition to adhesion molecules, other intercellular communication mechanisms have been shown to be involved in leukemia-MSC cross-talk. Interestingly, the formation of tunneling nanotubes (TNT) by B-ALL cells has been reported to actively stimulate MSCs to secrete CXCL10, CXCL8 and IL-2 [68]. Other contact-independent mechanisms, such as the release of EVs and other soluble mediators released by leukemic cells, such as inflammatory cytokines, that will be discussed later on, could further contribute to shape the chemokine milieu characterizing the leukemic BM niche.

Overall, these data suggest that deregulated chemokine axes could be exploited by leukemic cells to unseat healthy stem cells from BM protective niches and that their inhibition could offer valuable therapeutic targets to interfere with the BM niche in B-ALL.

#### 5.1.2. Altered Soluble Mediators of the TGFβ Family: Activin A, BMP4

The TGFβ family is a large group of structurally related proteins with a recognized role in embryological development and tissue homeostasis [74], more recently implicated in carcinogenesis, cancer progression, and microenvironment modification in several solid tumors [75,76] and hematological malignancies [77,78]. Alterations of the TGFβ pathway, which, as already mentioned, can favor the emergence of pre-leukemic cells over their normal counterparts, represent typical features also in the case of overt B-ALL, with BMP4 and Activin A resulting in the most involved molecules.

Interestingly, it has been published that ALL-MSCs isolated from BM aspirates at disease diagnosis and early phases of treatment (day+15) can produce higher BMP4 levels compared to HD-MSCs or ALL-MSCs isolated from out of therapy patients and that its production could be further induced by MSC co-culture with the t(12;21) B-ALL cell line REH [60]. BMP4 levels were inversely correlated with the proliferative ability of MSCs, which resulted impaired in ALL-MSC compared to HD-MSC [60]. Concerning the possible leukemia-promoting effects, it has been shown that BMP4 overexpression in ALL cells potentiates their ability to induce immunosuppressive dendritic cells (DCs) and to polarize macrophages to an M2-like pro-tumoral phenotype [79].

Another TGFβ family member deregulated within the B-ALL BM niche was Activin A. Our group recently described a significant increase in this molecule in the BM plasma of a large cohort of pediatric B-ALL patients, compared to HDs, and a higher Activin A production in ALL-MSCs, compared to HD-MSCs [63]. The production of this molecule was specifically driven by leukemia, since primary B-ALL cells induced a significant increase in Activin A release upon co-culture with HD-MSCs. Interestingly, as described in the previous paragraph, Activin A was demonstrated on the one hand to directly act on leukemic cells by enhancing their migratory and invasive properties, both in vitro and in a xenograft mouse model [63], and on the other hand by down-modulating CXCL12 production by HD-MSC [50].

Overall, the intimate cross-talk between leukemia and MSCs, resulting in in vitro TGFβ signaling alterations, suggests the cruciality of further in vivo studies about the impact of TGFβ targeting agents on leukemia progression and response to therapy.

#### 5.1.3. MSC-Induced Chemoresistance and Metabolic Exchanges with B-ALL Cells

The concept that cell-to-cell interaction with stromal cells can significantly improve leukemia cell survival is nowadays well recognized [80]. Several adhesion molecules have been implicated in the release of survival signals to the leukemic cell, thus mediating resistance to chemotherapy agents. The α4 integrin VLA-4 has been described to be highly expressed on several hematopoietic cells, including B-ALL blasts, and its overexpression on leukemia cells at first relapse has been correlated with poor molecular response to therapy and disease outcome [81]. One of the VLA-4 partners, VCAM-1, is instead expressed at high levels on the MSC membrane. Jacamo and colleagues demonstrated both in vitro and in vivo that leukemia-stroma interaction, through VCAM-1/VLA-4 binding, leads to the reciprocal activation of nuclear factor kappa B (NF-kB) mediated molecular pathways, necessary for mediating chemoresistance [82]. Interestingly, genetic or chemical inhibition of NF-kB signaling in BM-MSCs efficiently restored sensitivity to vincristine in B-ALL cells [82]. Furthermore, VCAM-1 blocking antibodies were also able to restore chemosensitivity to cytarabine and etoposide in MSC-leukemia co-cultures [83], suggesting that NF-kB or VLA-4/VCAM-1 targeting could be a clinically relevant mechanism to overcome stroma-mediated chemoresistance in B-ALL.

Cadherins are an ample family of transmembrane proteins with specific pro-tumoral or anti-tumoral roles, depending on the type. Their mutations and subsequent aberrant activation of the Wnt/β-catenin signaling have been implicated in chemoresistance in the context of several tumors [84]. Interestingly, Yang and colleagues [85] demonstrated that, upon interaction, BM stromal cells are able to protect ALL cells from Ara-C-induced apoptosis by inhibiting the cleavage and activation of apoptotic proteins such as PARP and Caspase-3, through the activation of the Wnt/β-catenin pathway. The usage of β-catenin inhibitor XAV939 enhanced Ara-C-induced apoptosis of leukemic cells co-cultured with MSCs and was able, in a mouse xenograft model, based on REH leukemia cell infusion, to enhance the survival of Ara-C treated mice [85]. Moreover, Wnt inhibition, by means of iCRT14 inhibitor, resulted in a further promising strategy to overcome chemotherapy resistance to prednisone and doxorubicin in B-ALL cells [86]. Interestingly, confocal analysis of t(1;19) B-ALL cells proved that N-cadherin guides the formation of adherent junctions with stroma cells thanks to the key action of β-catenin, which colocalized in the cell membrane along with N-cadherin, without the need for Wnt pathway activation [87]. Overall, these data suggest that cadherin-mediated interaction of MSCs with B-ALL cells and activated Wnt/β-catenin pathway could represent suitable targets for inhibitory drugs to improve chemosensitivity in B-ALL deserving further investigation.

Another pathway involved in MSC-mediated chemotherapy protection of B-ALL cells is the one based on Notch signaling, a developmental signaling pathway consisting of four receptors (Notch-1–4) and five ligands, including Jagged-1–2, DLL-1, and DLL-3–4. Among the variety of Notch receptors and ligands expressed both by B-ALL cells and MSCs, it has been demonstrated that Notch-3 and Notch-4 signaling specifically induced corticosteroids resistance in B-ALL cells co-cultured with BM-MSCs [88]. Interestingly, the usage of anti-Notch neutralizing antibodies or γ-secretase inhibitor XII, which prevents the release of cleaved intracellular active Notch, restored chemotherapy-induced apoptosis [89].

Galectin3 (*Lgals3*) is a multifunctional galactose-binding lectin highly expressed on the surface of MSCs, which can be released as soluble protein or as part of the cargo of MSC-derived exosomes [89]. It has been recently demonstrated that MSC-derived Galectin3 can be internalized by co-cultured ALL cells and further induce auto-production of endogenous Galectin3 in a sort of self-reinforcing loop. Interestingly, Galectin3 levels were found upregulated in vitro in nilotinib-resistant Ph-positive B-ALL cells and in leukemia cells harvested from a mouse after 8 days of drug treatment [90]. Moreover, it was demonstrated that MSC-induced Galectin3 promotes the activation of the Wnt/β-catenin signaling pathway, responsible, as already discussed, of B-ALL cell chemoresistance and that its silencing by the addition of Galectin3 short hairpin RNA (shRNA) could overcome the environmental-mediated drug resistance [91].

Several research groups have demonstrated that leukemic blasts and MSCs can actively exchange mitochondria [68,92] and that this transfer from activated MSCs could rescue B-ALL cells from apoptosis mediated by ROS-inducing chemotherapy [93]. MSC-derived mitochondrial transfer resulted in being dependent on TNT formation and was promoted by treatment with ROS-inducing agents such as cytarabine (Ara-C) and daunorubicin [93]. Interestingly, microtubule inhibitors, such as vincristine, could instead prevent MSC-mediated chemotherapy protection [93], suggesting that the biological effects of chemotherapy agents on the BM microenvironment should be carefully considered.

### 5.2. Interaction with Other Stromal Components: Bone Effects and Matrix Remodeling

Skeletal morbidities, including osteopenia and osteonecrosis, are acute and long-term invalidating burdens for B-ALL patients. Beyond being recognized as a treatment effect, the observation of bone fractures in children at B-ALL diagnosis suggests that leukemic cells could directly influence the formation and maintenance of bone matrix. Within a physiological BM niche, osteoblasts and osteoclasts maintain bone homeostasis by either promoting bone formation or resorption [94]. Furthermore, by cell-to-cell contact signals and the release of soluble mediators, such as G-CSF, they regulate, as already mentioned, the niche size and the number of HSCs.

Recent literature highlights that B-ALL cells isolated at patients’ diagnosis can cause bone destruction [95]. In detail, Rajakumar and colleagues showed that the transplant of primary B-ALL cells in NSG recipient mice induced trabecular bone destruction, associated with increased multinucleated osteoclasts and that the receptor activator of NF-kB (RANK)/RANK ligand (RANKL) axis was a key mediator of these effects. Indeed, the usage of an antagonist of RANKL, a recognized regulator of osteoclast differentiation, protected the bone from leukemia-induced destruction in patient-derived xenograft mice [95]. Leukemogenesis-related bone loss was further documented in a leukemia-bearing BCR-ABL1+ xenograft mouse model as a result of RANKL production by leukemic cells and osteoclast activation [96]. Furthermore, reduced osteoblastic cell number and collagen production were observed in this mouse model, suggesting a profound effect of B-ALL cells on the BM microenvironment [96].

Based on the above-mentioned role of osteoclasts and osteoblasts in regulating HSCs, the consequences of endosteal niche remodeling should be carefully evaluated. Leukemic cell interaction with both cell subsets could not be limited to the induction of skeletal abnormalities but could directly result in a perturbation of healthy hematopoiesis, suggesting that a prompt intervention with targeted therapies could reduce acute and long-term bone destruction in B-ALL patients and rescue the HSC pool.

### 5.3. Altered Molecular Pathways in the Cross-Talk between Vascular Cells and B-ALL Cells

Endothelial and mural cells (pericytes and smooth muscle cells) contribute, as already discussed, to hematopoiesis by providing a specialized “vascular niche” for HSCs, where HSC self-renewal and expansion or differentiation processes are tightly regulated, depending on secreted soluble factors [97,98]. Moreover, blood vessels are key components in the pathogenesis of tumors for different reasons. Firstly, rapidly growing cells necessitate a continuous flow of oxygen and other nutrients, such as essential amino acids, oligoelements etc., for the biogenesis of cellular components and for cell division. Secondly, they participate in tumor progression by representing highways for cancer spread around the body and metastatization.

Recent evidence suggests that B-ALL cells can impact BM vascularization. In a recent study to explore the effect of B-ALL progression on a steady-state BM, primary cells were transplanted from the lymph node of a leukemic *Cd45.2^+/+^Ebf1^+/^Pax5^+/−^* mouse to wild-type Cd45.2 mice by tail vein injection, without pre-conditioning [99]. Interestingly, results from this mouse model suggested leukemia-driven changes in the vascular composition and functionality, with an increased fraction of CD31+ endothelial cells at early time-points after leukemic cell transplantation [99]. Accordingly, in a large cohort of children with acute leukemia, it was demonstrated that microvessel density (MVD) at disease presentation is significantly increased compared to age-matched controls or remission biopsies, but this value did not show any prognostic or predictive value [100]. MVD increase was further confirmed in a cohort of adult ALL patients [101]. Angiogenic factors, including VEGF and bFGF, have also been investigated in terms of concentrations and correlation with clinical outcome, by several groups. Unfortunately, obtained results are far from being conclusive since they are contradictory, depending on the type of sample analyzed (urine, PB, BM plasma) and the cohort investigated [102]. Indeed, well planned studies on large cohorts of age-matched patients are needed to clarify this point and to identify angiogenic molecules with a potential prognostic or predictive value, that can be secreted either by leukemic cells or by leukemia-reprogramed vascular cells.

Concerning the ability of B-ALL cells to induce changes in the secretome of vascular cells, we recently demonstrated [70] that B-ALL cells upon co-culture with human umbelical vein endothelial cells (HUVEC) are able to significantly increase their secretion of CCL2, whose role in the B-ALL niche has been already highlighted. Moreover, we also demonstrated that inflammatory cytokines, abundant in the B-ALL BM niche, can impact chemokine production by vascular cells, such as, for example, increasing the release of CCL2 and CX3CL1 by HUVEC cells [70]. The fact that leukemic cells could modulate chemokine production by vascular cells is particularly intriguing in view of the key role of blood vessels in the recirculation of immune cells to the BM niche, where they can become dangerous allies of leukemic cells, as discussed in the following paragraph.

Thanks to the highly vascularized BM microenvironment, the initial bulk of proliferating B-ALL cells originating in the BM can disseminate into extramedullary sites. After gaining access to BM blood vessels, leukemic cells can extravasate through a process called transendothelial migration, which requires cytoskeletal remodeling into peripheral tissues such as the liver, testis, central nervous system (CNS) etc. [103]. It has been recently demonstrated that B-ALL cells expressing high levels of the actin-binding protein cortactin show the highest levels of CXCL12-driven transendothelial migration and, in xenotransplantation models, were the only cells infiltrating lung, brain, and testis [104]. Another molecule that has been correlated with increased transendothelial migration and in vivo dissemination of B-ALL leukemic cells is myosin-IIA. Indeed, inhibition of this cytoskeletal class II myosin motor protein significantly decreased CNS infiltration of leukemic cells in a leukemia mouse model [103]. These data suggest that the identification of key molecules in the interaction of B-ALL cells with vascular cells would be crucial for identifying new therapeutic targets to limit leukemic dissemination.

### 5.4. Deregulation of Immunity-Related Components within the B-ALL Microenvironment

Inflammatory mediators, innate and adaptive immune cells cooperate to create a tumor-permissive microenvironment. As a general paradigm, tumors behave as a never-healing wound, where inflammatory cells contribute to an immunosuppressive tumor microenvironment [105]. In the context of B-ALL, the contribution of immune cells to the leukemic microenvironment is far from being elucidated but has become, in the last decades, a very active field of research with promising results in terms of clinically translational targets to improve the management of the disease.

#### 5.4.1. Monocytes and Macrophages

Myelomonocytic cells are a heterogeneous subset of immune cells, displaying distinct functions in response to different stimuli, that have emerged as key regulators of solid cancer development and progression. Furthermore, their contribution to the pathogenesis of hematological cancers has clearly emerged. Several studies in the field of chronic lymphocytic leukemia (CLL) have highlighted the presence in the BM microenvironment of nurse-like cells, which are tumor-associated macrophages, with a critical role in the survival and chemoresistance of CLL cells [106,107,108,109]. In the context of B-ALL, a first paper suggested that leukemic cells can condition monocytes to an inflammatory phenotype, able to promote migration and invasive properties of B-ALL cells [110]. More recently, Witkowski and colleagues demonstrated significantly lower overall survival and relapse-free survival in pediatric B-ALL patients presenting with absolute PB monocytosis at disease diagnosis, independent of other risk factors [111]. Moreover, by a combination of single-cell RNA sequencing and cellular indexing of transcriptomes and epitopes (CITE) sequencing, they demonstrated that increased monocytes in the BM of B-ALL patients at diagnosis and relapse are prevalently CD14dimCD16+CD115+ non-classical monocytes [111]. Finally, by using a mouse model of pediatric Ph+ B-ALL, they recapitulated the emergency of a CD11b+CX3CR1+Ly6C− murine cell population reminiscent of the non-conventional human (NC) monocytes. Interestingly, the delivery in this mouse model of anti-CSF1R antibodies, which depletes monocytes by inducing apoptosis and blocking their differentiation to macrophages, along with the tyrosine kinase inhibitor nilotinib, significantly increased the overall survival of leukemia-transplanted mice [111]. In line with their findings, our group confirmed the increase in NC CD14dimCD16+ monocytes in the PB of B-ALL patients at diagnosis, compared to controls, and highlighted that they express low levels of the CCL2 receptor CCR2, but very high levels of the Fractalkine (CX3CL1) receptor CX3CR1 [70], as previously shown for this monocyte subset under physiological conditions [112]. Interestingly, we also demonstrated for the first time a significant increase in CX3CL1 in the BM plasma of pediatric patients at B-ALL diagnosis compared to age-matched controls, suggesting that the CX3CL1/CX3CR1 could represent a novel chemokine axis crucial for the recruitment of NC monocytes to the leukemic BM niche [70]. Importantly, CX3CL1/CX3CR1 has been already described as a deregulated pathway in the context of several tumors such as CLL and multiple myeloma, with a crucial role in the cross-talk between cancer cells and tumor microenvironment [113,114].

The concept that the myeloid compartment is highly remodeled at B-ALL diagnosis is further supported by data coming from the study of leukemia-associated macrophages. In this regard, we recently demonstrated an increased quote of cells expressing the macrophage marker CD68 in BM biopsies obtained from B-ALL patients at disease diagnosis, compared to controls [70]. Interestingly, B-ALL-associated macrophages mainly expressed the typical M2-like polarization markers CD163 and CD206 [70]. These findings are in line with data from CLL, where macrophages expressing an M2-like immunosuppressive phenotype were able to support the survival and proliferation of CLL cells and to protect them from drug-induced apoptosis [107,115]. It is conceivable to think that inflammatory monocytes recruited into the leukemic BM niche would be reprogrammed by surrounding cells, including leukemic cells, to become leukemia-supporting. In favor of this hypothesis, it has been demonstrated that CCL2, whose levels were significantly increased in the B-ALL BM niche [70], has a crucial impact on the polarization of human macrophages to an M2-like, tumor-associated phenotype [116,117]. Furthermore, it has been recently demonstrated that BMP4 overexpression in ALL cells potentiates their ability to induce immunosuppressive DCs and to promote the generation of M2-like macrophages with pro-tumoral features [79]. Further studies on the mechanisms of macrophage recruitment and polarization in the context of B-ALL will be essential to identify potential targetable pathways.

A comprehensive analysis of B-ALL BM biopsies by means of multiplexed immunohistochemistry further confirmed the specific decrease in M1-like macrophages, along with an increased proportion of M2-like macrophages [118]. In addition, granzyme B+CD57+CD8+ T cells, and CD27+ T cells resulted decreased, while myeloid-derived suppressor cells and PD1+TIM3+ double-positive CD4+ T cells were significantly upregulated, with the latter being independent predictors of poor outcome in a multivariate risk model [118]. Overall, these data suggest that the immune cell contexture in ALL BM widely differs from healthy controls, not only in terms of monocytes and macrophages, as thereafter described.

#### 5.4.2. Myeloid-Derived Suppressor Cells (MDSCs), Regulatory T Cells (Tregs), and Dendritic Cells (DCs)

MDSCs have emerged as important contributors to tumor expansion and chronic inflammation progression by inducing immunosuppressive mechanisms, angiogenesis, and drug resistance.

Recent evidence suggests that MDSC could take part in the definition of the malignant leukemic microenvironment. Several studies have shown an increased number of MDSC in the BM and PB of B-ALL patients compared to controls [118,119]. Interestingly, their number resulted modulated by induction chemotherapy, suggesting a possible contribution in mediating drug resistance to leukemic cells [120]. In addition, the frequency of MDSCs belonging to the granulocytic subset (G-MDSC) resulted positively correlated with prognostic markers of therapy response, such as minimal residual disease (MRD) and with blast cell counts [119]. Interestingly, studies on the expansion of MDSCs in the context of myelodysplastic syndrome (MDS), showed a high membrane expression of CX3CR1 and CXCR4 on patients MDSCs compared to healthy controls and that these levels were correlated with patients’ risk stratification [121]. These data suggest, in view of the increased CX3CL1 levels observed in the BM plasma of B-ALL patients [70], that CX3CL1/CX3CR1 should be investigated as a possible chemokine axis guiding the recruitment not only of NC monocytes (see above), but also of MDSCs in the leukemic BM niche.

T cells are physiologically present in peripheral tissues where they exert their effector functions necessary to eradicate infected cells but also to control the outgrowth of cancer cells. A high number of studies in the context of solid tumors proved that patients’ prognosis strictly depends upon the ratio between effector T cells and regulatory T cells, the latter endowed with immunosuppressive properties associated with tumor protection. Literature data suggest that this paradigm could be relevant also in the context of B-ALL. Interestingly, high CD4/CD8 ratios at diagnosis correlated with a favorable BM response on day 15 only if non-T(reg) CD4+ cells were taken into account [122]. Concerning the Treg subset, several studies demonstrated increased frequencies in the PB and BM of B-ALL patients compared to controls [120,123,124]. Recently, immunosuppressive functions of Tregs in B-ALL patients have been correlated with the expression of the transcription factor Helios in FoxP3+ CD4+ Tregs [125]. Indeed, Helios was able to regulate the production of TGFβ by Tregs and to modulate angiogenesis in the BM niche of pre-B-ALL via the VEGFA/VEGFR2 pathway [125].

Interestingly, the frequency of Tregs has been recently shown to impact response to therapy in refractory/relapsed (r/r) B-ALL patients, treated as salvage therapy with the T-cell-engaging, anti-CD19 bispecific antibody blinatumomab [126]. In this study, it was demonstrated that blinatumomab non-responders had a significantly higher number of Tregs that, activated by the bispecific antibody, released IL-10, thus causing reduced T-cell proliferation and CD8-mediated lysis of ALL cells [126]. Furthermore, depletion of Tregs from PB patients’ samples, restored in vitro the blinatumomab-triggered proliferation activity of r/r ALL patient T cells [126]. These data suggest that coupling anti-microenvironment with anti-leukemia strategies represents a real chance for improving patients’ response and decreasing the frequency of relapse.

DCs are professional antigen-presenting cells, with a key role at the cross-road between innate and adaptive immunity. Numerical as well as maturation-associated alterations have been described in the context of several solid tumors. Scarce data are available for B-ALL. However, published reports support the idea that a general impairment of DC function could be relevant for the poor anti-leukemia immunity in B-ALL pathology.

A few groups concordantly demonstrated that the numbers of both myeloid (mDC) and plasmacytoid (pDC) DCs were significantly reduced at B-ALL diagnosis compared to age-matched controls [127,128]. This alteration was typical of B-ALL since T-ALL patients, on the contrary, showed comparable or even increased DC numbers. Furthermore, experiments showing that circulating CD34+ cells from B-ALL patients fail to differentiate in vitro, under the appropriate stimuli, in both mDC and pDC, suggest that an altered DC development is likely to be responsible for the observed numerical deficit [128]. More recently, it was demonstrated that the conditioned medium from the B-ALL cell line NALM-6 inhibited in vitro monocyte differentiation to CD1a high CD14–/low DCs [79]. Interestingly, this deficit was associated with BMP4 production by the B-ALL cells, and the overexpression of this molecule in NALM-6 cells potentiated their ability to induce immunosuppressive DC and the generation of M2-like macrophages with pro-tumoral features [79].

#### 5.4.3. Inflammatory Mediators

As previously mentioned, inflammatory mediators exert a key role in generating a tumor-promoting microenvironment. It is well known that oncogenes and altered suppressor genes can activate inflammation-related gene cascades. Moreover, external factors of microenvironment origin can further contribute to promoting prolonged inflammatory responses. Cellular components of cancer-related inflammation, including neutrophils, macrophages, and soluble mediators, such as inflammatory cytokines, Complement cascade components, and their regulators, can favor leukemia maintenance and progression by regulating angiogenesis and metastasis, promoting genetic instability and chemoresistance, and suppressing immunity [105].

In the case of B-ALL, it has been documented that leukemic cells themselves have the ability to produce and secrete inflammatory factors, including TNFα, IL-1β, and IL-12, thus contributing to the definition of a pro-inflammatory microenvironment that may be detrimental to long-term normal hematopoiesis [129].

Among these factors, it has been recently proven, by means of in vitro and in vivo assays, that B-ALL-derived TNFα can activate NF-kB downstream of tumor necrosis factor receptor 1 (TNFR1) on MSCs, and possibly on other cell subsets within the BM niche, resulting for example in matrix metallopeptidase 9 (MMP-9) secretion [130]. Moreover, we recently demonstrated that the stimulation of MSCs and endothelial cells by a cocktail of the inflammatory cytokines TNFα, IL-1β and IL-6, that we found upregulated in the BM plasma of B-ALL patients, was able to modify the production and secretion of several chemokines such as CCL2 and CX3CL1, whose possible role has been already discussed [70].

Beyond B-ALL cells, MSCs can further contribute to the release of inflammatory mediators in the BM niche. It has been demonstrated that MSCs isolated from the BM of B-ALL patients have increased nuclear translocation of the NF-kB transcription factor and consequent increased production of pro-inflammatory mediators including IL-1α, IL-6, IL-12p70, and TNFα, as well as interferon type I and type II [64]. An innovative study taking advantage of a unique 3D organotypic “leukemia-on-a-chip” microphysiological system that recapitulates the architecture and the microenvironment-leukemia interactions characterizing the BM leukemic niche further supported the idea that leukemia progression is strictly linked with the activation of an inflammatory program [131]. Seeding and growth of different B-ALL cell lines in this leukemia BM niche model showed a progressive production of chemokines such as CCL2 and pro-inflammatory cytokines, including IL-6 and CXCL8 [131]. Moreover, the authors demonstrated that a significantly enriched gene expression profile related to TNFα signaling via NF-kB and inflammation response activation were associated with most of the cell types, suggesting the evolution of an inflammatory scenario upon leukemia progression [131]. Indeed, direct leukemia-MSC contact via VCAM-1/VLA-4 interactions [82], together with CXCL12/CXCR4 signaling and inflammatory cytokine signaling [131,132], may contribute, to a different extent, based on space and time, to enhance leukemia survival by activating NF-kB signaling within the leukemic BM niche.

In the context of several solid tumors, it has been demonstrated that the epigenetic repression of the Complement cascade regulator PTX3 in the tumor microenvironment is strictly linked to induction of complement-dependent tumor-promoting inflammation [133]. Our group demonstrated, for the first time, an increased C5a fraction along with decreased PTX3 levels in the BM plasma of B-ALL patients, compared to controls and showed by co-culture experiments that B-ALL cells could directly interfere with PTX3 production by BM-MSCs [70]. Interestingly, literature data indicate that C5a could play a leukemia-promoting role by influencing, in concert with C3a, migratory and adhesive properties of leukemia cells by downregulating HO-1, an inducible enzyme with anti-inflammatory functions [134]. Furthermore, as already demonstrated in the context of colon cancer [135], C5a could support local chronic inflammation and hamper antitumor T-cell responses by recruiting, in concert with CCL2, leukemia-associated macrophages and promoting their polarization to a M2-like phenotype.

## 6. Conclusions

External cellular and molecular perturbations of the healthy hematopoietic BM niche provide fertile soil for the leukemic transformation of the pre-leukemic clones. Epidemiological studies and experimental evidence suggest that infection-related inflammation represents a crucial trigger event through the creation of a genotoxic microenvironment, which facilitates the accumulation of DNA mutations in apoptosis-resistant pre-leukemic cells. Under these leukemia-favoring conditions, the pre-leukemic phase can progress to overt leukemic disease. Pre-leukemic cells can also find a permissive context to survive multi-chemotherapy and re-emerge at disease relapse. Indeed, the bulk of accumulating leukemic cells continuously cross-talk with the surrounding microenvironment creating an altered BM niche, which promotes their survival, chemoresistance, and progression at the detriment of healthy hematopoietic progenitors. Overall, intrinsic and extrinsic pathways could contribute to sustaining leukemia-related inflammation characterizing all the phases of B-ALL and could offer different complementary targets that could be relevant to restore a BM microenvironment less favorable to disease onset and progression.

Based on this evidence, a therapeutic strategy including both a direct targeting of pre-leukemic/leukemic cells and altered pathways crucially involved in their cross-talk with the BM niche represents an essential goal for achieving better disease management and possibly its prevention.

In this regard, therapeutic strategies against immunosuppressive populations (e.g., tumor-associated macrophages, Tregs etc.), leukemia-supporting molecules expressed by stroma cells, altered cytokine networks and immune checkpoint signals are under investigation in several solid and hematological malignancies [136,137]. At this purpose, chimeric receptor antigen (CAR)-T cells, antibodies against developmental pathways (e.g., anti-CSF1R antibody) [111], neutralizing antibodies for soluble factors [44] (e.g., inflammatory cytokines, BMP4, Activin A etc.) could be harnessed to target the “corrupted” leukemia microenvironment in combination with chemotherapy-based or immune-mediated anti-leukemia strategies. Microenvironment/leukemia dual targeting would result into a better disease management by increasing leukemic cell chemosensitivity and improving the effectiveness of tumor-directed immunotherapy approaches, including anti-leukemia CAR-T cells.

Concerning the pre-leukemic phase, therapeutic strategies are still far from clinical application. However, microbiome manipulation has been hypothesized as an innovative approach for leukemia prevention. The idea is that oral administration of benign symbiotics could mimic the protective impact of natural infections during infancy, thus educating the immune system and lowering the possibility to develop the disease in genetically predisposed children [4,138].
